# Transcription Factor-MicroRNA-Target Gene Networks Associated with Ovarian Cancer Survival and Recurrence

**DOI:** 10.1371/journal.pone.0058608

**Published:** 2013-03-12

**Authors:** Kristin R. Delfino, Sandra L. Rodriguez-Zas

**Affiliations:** 1 Department of Animal Sciences, University of Illinois, Urbana, Illinois, United States of America; 2 Department of Statistics, University of Illinois, Urbana, Illinois, United States of America; 3 Institute for Genomic Biology, University of Illinois, Urbana, Illinois, United States of America; University of Illinois-Chicago, United States of America

## Abstract

The identification of reliable transcriptome biomarkers requires the simultaneous consideration of regulatory and target elements including microRNAs (miRNAs), transcription factors (TFs), and target genes. A novel approach that integrates multivariate survival analysis, feature selection, and regulatory network visualization was used to identify reliable biomarkers of ovarian cancer survival and recurrence. Expression profiles of 799 miRNAs, 17,814 TFs and target genes and cohort clinical records on 272 patients diagnosed with ovarian cancer were simultaneously considered and results were validated on an independent group of 146 patients. Three miRNAs (hsa-miR-16, hsa-miR-22*, and ebv-miR-BHRF1-2*) were associated with both ovarian cancer survival and recurrence and 27 miRNAs were associated with either one hazard. Two miRNAs (hsa-miR-521 and hsa-miR-497) were cohort-dependent, while 28 were cohort-independent. This study confirmed 19 miRNAs previously associated with ovarian cancer and identified two miRNAs that have previously been associated with other cancer types. In total, the expression of 838 and 734 target genes and 12 and eight TFs were associated (FDR-adjusted P-value <0.05) with ovarian cancer survival and recurrence, respectively. Functional analysis highlighted the association between cellular and nucleotide metabolic processes and ovarian cancer. The more direct connections and higher centrality of the miRNAs, TFs and target genes in the survival network studied suggest that network-based approaches to prognosticate or predict ovarian cancer survival may be more effective than those for ovarian cancer recurrence. This study demonstrated the feasibility to infer reliable miRNA-TF-target gene networks associated with survival and recurrence of ovarian cancer based on the simultaneous analysis of co-expression profiles and consideration of the clinical characteristics of the patients.

## Introduction

Ovarian cancer, the most malignant gynecologic neoplasm, is the fifth leading cause of cancer deaths among women. Approximately 45% of ovarian cancer patients survive more than five years after initial diagnosis and less than 20% surpass this milestone once the cancer has disseminated [1]. Few gene expression profiles have been consistently related to ovarian cancer [2], [3]. This may be due to the limited simultaneous consideration of the transcripts and transcript regulators associated with ovarian cancer.

MicroRNAs (miRNAs) are small, non-coding RNA molecules that bind to complementary sequences on target mRNA transcripts, and thus, regulate gene expression at the post-transcription stage. Transcription factors (TFs) are a different type of regulator. These proteins bind to specific DNA sequences in the promoter region, promoting or repressing transcription into mRNA, and thus, regulate genes at a pre-transcription stage [4]. TFs and miRNAs can regulate each other and both can regulate the expression of target genes. TF-miRNA-target genes can function as onco or tumor suppressor networks, triggering global alterations of genetic programs implicated in cell proliferation, differentiation, apoptosis, and invasiveness in cancer.

Few associations between ovarian cancer and miRNAs or TF have been validated in independent studies [2], [3]. Several reasons may be behind the limited understanding of the regulatory networks associated with ovarian cancer. First, most studies associate ovarian cancer to genes (miRNAs or TFs) on an individual basis instead of considering multiple profiles simultaneously. Second, even when studies analyze multiple genome profiles simultaneously, the relationship between target genes and regulatory miRNAs and TFs are not used. Third, most studies do not consider clinical or cohort-dependent factors when characterizing associations between expression profiles and ovarian cancer. Lastly, most studies consider the binary qualitative trait presence or absence of cancer, and more quantitative measurements such as survival and recurrence are not evaluated.

The main objectives of this study were a) to develop a model to identify and characterize miRNAs, TFs, and target genes associated with ovarian cancer survival, and b) use this information to identify TF-miRNA-target gene networks associated with survival in ovarian cancer. Our overarching hypothesis was that reliable gene expression biomarkers of cancer can be obtained from the consideration of all components in a network simultaneously. A systems biology approach was used to investigate the simultaneous association between multiple miRNAs, TFs, and target genes and cancer survival or recurrence, accounting for non-genetic patient-to-patient sources of variation, and the corresponding networks were analyzed. Results were validated in an independent data set. The study also identified enriched functional categories and pathways of genes associated with cancer survival and recurrence. Understanding the molecular basis of ovarian cancer is key to developing improved prognostic indicators and effective therapies. Given the heterogeneity of this disease, improvements in long-term survival might be achieved by translating recent insights at the molecular and clinical levels into personalized individual treatment strategies.

## Materials and Methods

### Training Data Set

#### Clinical information

Survival, recurrence, cohort, and genomic expression information from 272 patients diagnosed with ovarian cancer was obtained from The Cancer Genome Atlas (http://cancergenome.nih.gov/) repository (Accessed September 2009) [5]. Cohort factors analyzed include treatment received (only chemotherapy, 93%; chemotherapy plus another treatment, 5%; and any treatment other than chemotherapy, 2%); preadjuvant therapy (yes, 8% or no, 92%); additional treatment (only chemotherapy, 41%; chemotherapy plus another treatment, 14%; and any treatment other than chemotherapy, 45%); tumor stage (stage I or II, 4%; stage III, 88%; stage IV, 8%); tumor grade (grade I or II, 4%; any grades other than I or II, 96%); tumor residual disease (no macroscopic disease, 26%; 1–20 mm, 61%; greater than 20 mm, 13%); recurrence (yes, 58% or no, 42%), and age at diagnosis (in years). Preadjvant therapy refers to any treatment that the patient received prior to surgery and sample collection. Tumor stage refers to the pathological stage of the tumor in AJCC format (Primary Tumor: T; Stage 1∶1A; 1B; 1C; Stage II: IIA; IIB; IIC; Stage III: IIIA; IIIB; IIIC; Stage IV: IV). Tumor grade is the numeric value used to express the degree of abnormality of cancer cells and is a measure of differentiation and aggressiveness. Tumor residual disease is the measure of the largest remaining nodule. Age refers to the age in years of the individual at the time of diagnosis of ovarian cancer. These cohort factors were accounted for in the analysis because of their known association with survival [6].

#### Expression profiling

The expression levels of 799 miRNAs were measured using the Agilent 8 × 15K Human microRNA platform (Agilent Technologies, http://www.genomics.agilent.com/). The expression levels of 17,814 TFs and target genes were measured using the Agilent Custom Gene Expression G4502A_07 human gene platform. The transcriptome data is available at (https://tcga-data.nci.nih.gov/tcga/dataAccessMatrix.htm). The expression measurements were quantile normalized (probe level), collapsed within the miRNA, TF or gene, and log_2_ transformed following the procedures available in Beehive (http://stagbeetle.animal.uiuc.edu/Beehive) [7] and previously described in [8]–[10].

### Model and Profile Selection

Two ovarian cancer response variables were studied: 1) survival time from diagnosis to death (months from diagnosis to death); and 2) recurrence time from diagnosis to recurrence (months from diagnosis to recurrence). Information on comorbidities or cause of death was unavailable, thus the first variable describes the time-dependent likelihood of death, conditional on a prior ovarian cancer diagnostic, irrespective of cause of death or comorbidity. An ovarian cancer predictive model that simultaneously considered all miRNAs and cohort information was used to identify general (or cohort-independent) and personalized (or cohort-dependent) biomarkers. This model overcame limitations of prior studies which ignored the simultaneous association by only analyzing one miRNA at a time or ignoring possible cohort relationships.

A biomarker identification pipeline was implemented based on the multivariate Cox survival analysis and complementary feature selection strategies [8], [9], [11]. The Cox proportional hazard model assumes a parametric model to test the association between the covariates and the hazard ratio (HR) of the event. After transformation, the hazard (instant probability) of event (death or recurrence) was modeled with a linear combination of a baseline hazard and explanatory covariates including all the cohort variables, the expression profiles of all genome variables (miRNAs, TFs, or gene targets), and the interaction between them [12]. Stepwise and forward selection strategies were used to identify the expression profiles associated with survival or recurrence because of the complementary advantages of these strategies. Profiles remained in the hazard predictive model after consideration of other biomarkers at P-value <0.1. The significant profiles from the previous stepwise and forward model were included in a model that was subjected to a stepwise selection. This step allowed the identification of broad or general associations between profiles and ovarian cancer hazards that can be used as population prognostic biomarkers. The relaxed P-value threshold allowed detection of profiles that may have weak associations among large sets of profiles and stronger associations as the set was streamlined. In the second step, the interaction between the selected profiles and cohort indicators were evaluated using the stepwise approach. This step allowed the identification of cohort-dependent associations between profiles and the hazard of ovarian cancer death or recurrence that can be used as individualized predictive biomarkers. In the third step, all selected profiles and interactions were combined and further streamlined using the stepwise method. The association between the ovarian cancer hazards and the cohort factors and expression profiles was visualized by plotting the probability of survival predicted from the Cox model against time.

The test of no association between the miRNA, TF, gene or cohort prognostic markers and the HR between cohort groups and the 95% confidence interval limits follow a Chi-square distribution. Hazard ratio estimates >1 (<1) indicate an increase in the hazard (decrease in the hazard) or decrease in survival probability (increase in survival probability) per unit increase in the level of gene expression. A False Discovery Rate (FDR)–adjusted P<0.05 and |HR/expression unit| >1.15 thresholds were used to identify molecular factors associated with ovarian cancer survival or recurrence. The analysis was implemented using PROC PHREG in SAS [13].

The Cox model assumes proportional hazards across the period studied. This assumption can be expressed as parallel survival functions across the levels of expression profiles or cohort variables in the model. This assumption was tested for the two hazards considered, and there was no indication of significant departure from the assumption. Furthermore, visualization of the survival and residuals did not suggest departure from the model assumptions. There was no indication of significant departure from the proportional hazards assumption, also confirmed by the overlap on miRNAs, TFs, and genes between survival indicators. Biomarkers identified in this study were searched against the ovarian cancer and cancer literature based on independent data sets.

### Functional Enrichment and miRNA-TF-target Gene Networks

The known and predicted relationships between miRNAs, TFs, and target genes were obtained from the MIR@NT@N resource (http://maia.uni.lu/mironton.php, [14]). Only the relationship between transcription factors, miRNAs, and target gene supported by a mapping score >0.85 that correspond to a median P-value <1×10^−3^ and 90% of the relationships with P-values <1×10^−2^ were considered. The enrichment of Gene Ontology (GO, http://www.geneontology.org/) [15] molecular functions and biological processes and KEGG (http://www.genome.jp/kegg/) [16], [17] pathways was studied among the target genes associated with ovarian survival and recurrence. Two functional analyses were evaluated. The first functional analysis consisted on Fisher’s exact (two-tailed) test implemented in DAVID v6.7 (http://david.abcc.ncifcrf.gov/) [18] was used to identify the functional categories enriched among all target genes associated (FDR-adjusted P-value <0.05) with survival or recurrence [8], [9]. Categories that had at least 5 genes and were significant at FDR-adjusted P-value <0.1 were considered enriched. This analysis offered a baseline understanding of the categories associated with ovarian cancer.

The second functional analysis consisted on a set enrichment analysis [19] of all target genes regardless of the significance level of the association with ovarian cancer survival or recurrence. This analysis considered the association between survival or recurrence and gene expression through the sorting of the target genes by the magnitude, sign, and standard error of the estimate in the underlying scale or log_e_(HR). Positive estimates correspond to HRs >1 and thus lower survival or higher risk of recurrence. Conversely, negative estimates correspond to HRs <1 and thus higher survival or lower risk of recurrence. The set enrichment analysis implemented in Babelomics v4.3 (http://babelomics.bioinfo.cipf.es/) [19] was used to apply a segmentation test that identifies for asymmetrical distributions of functional categories between the genes ranked from negative to positive log_e_ (HR) estimates for ovarian cancer survival or recurrence. Categories significant at FDR-adjusted P-value <0.05 and having at least 75 genes were considered enriched. The less stringent threshold used for the Fisher’s enrichment analysis relative to the set enrichment analysis was motivated by the higher number of target genes analyzed in the second analysis relative to the first analysis. The genes associated with ovarian cancer hazard were also searched against the Dragon database of ovarian cancer genes (http://apps.sanbi.ac.za/ddoc/) [20]. The networks of TFs, miRNAs, and target genes significantly associated with ovarian cancer survival or recurrence (P-value <0.01) were depicted using Cytoscape (http://www.cytoscape.org/) [21], an open source software platform for visualizing networks and including attributes. The distribution and connectivity of the TFs, miRNAs, and target genes within sub-networks and the overall network were characterized.

### Validation Data Set

The associations between expression profiles and ovarian cancer survival or recurrence identified based on P-values and characterized based on HR estimates were validated on an independent data set of 146 patients obtained from the TCGA repository. Two indicators of the reliability of the predictive profiles in the independent validation were considered. First, mean square error (MSE) was used as measure of the lack of adequacy of the cohort-independent and -dependent expression profiles to accurately predict the time to death or recurrence in the training and validation data sets. Second, additional validation of the detected profile association was gained from the study of the correlation of the estimates (log_e_(HR)) corresponding to each profile between training and validation data sets. The Pearson and Spearman correlations of the profile associations with death and recurrence between the training and validation data sets were computed.

## Results and Discussion

**Table 1** summarizes the number and distribution of individuals studied across levels of the cohort covariates considered in the training and validation data sets. The median age at diagnosis was 60.2 years and 59.6 years for the training and validation data sets, respectively. These were consistent with the National Cancer Institute reports that the median age at diagnosis for cancer of the ovary (from 2004–2008) was 63 years of age and the median age at death was 71 years of age [22]. The range of age at diagnosis was 57 years (ages from 27 to 84 years) and 52 years (ages from 37 to 89 years) for the training and validation data sets, respectively. The median time for survival and recurrence for the training set was 2.4 years and 47.4 months and for the validation set were 3.3 years and 58. 7 months, respectively. The Pearson and Spearman correlation coefficients between both events (age at death and at recurrence) were 0.72 and 0.77 (P-value <0.0001), respectively in the training data set and 0.69 and 0.68 (P-value <0.0001), respectively in the validation data set. These statistics were in agreement with previously documented survival rates of ovarian cancer: 1 year: 77.5%, 2 year: 64%, 3 year: 54.4%, 5 year: 43.9%, 8 year: 37.8%, 10 year: 36.4% [22]. Median survival for patients was 25.7 months for early treatment patients and 27.1 months for those patients in a delayed treatment group [22].

**Table 1:** Number and distribution of individuals analyzed for post-diagnostic survival and post-diagnostic recurrence and levels of the cohort factors considered.

		Survival				Recurrence			
		Training Set		Validation Set		Training Set		Validation Set	
		Number	Percent	Number	Percent	Number	Percent	Number	Percent
**Total**		272		146		157		92	
**N** 1 **Censored**		107	39%	75	51%	31	20%	38	41%
**Treatment** 2	*Chemo* 3	253	93%	101	69%	150	96%	67	72%
	*Chemo_Other* 4	14	5%	25	17%	5	3%	18	20%
	*Other* 5	5	2%	20	14%	2	1%	7	8%
**Preadjuvant Therapy** 6	*Yes*	21	8%	36	25%	7	4%	25	27%
	*No*	251	92%	110	75%	150	96%	67	73%
**Additional Treatment** 7	*Chemo* 3	113	41%	49	34%	106	67%	45	49%
	*Chemo_Other* 4	37	14%	35	24%	34	22%	35	38%
	*Other* 5	122	45%	62	42%	17	11%	12	13%
**Tumor Stage** 8	*I_II* 9	12	4%	17	12%	5	3%	9	10%
	*III* 10	239	88%	89	61%	143	91%	59	64%
	*IV* 11	21	8%	40	27%	9	6%	24	26%
**Tumor Grade** 12	*I or II* 13	12	4%	45	31%	6	4%	35	38%
	*Rest* 14	260	96%	101	69%	151	96%	57	62%
**Tumor Residual Disease** 15	*0* 16	71	26%	52	36%	34	22%	30	33%
	*1_20* 17	167	61%	60	41%	102	65%	40	43%
	*>20* 18	34	13%	34	23%	21	13%	22	24%
**Recurrence** 19	*Yes*	157	58%	92	63%	157	100%	92	100%
	*No*	115	42%	54	37%	0	0%	0	0%

1 N: Number of patients;

2 Treatment: Type of treatment received;

3 Chemo: Only chemotherapy;

4 Chemo_Other: Chemotherapy plus another treatment;

5 Other: Any treatment other than chemotherapy;

6 Preadjuvant Therapy: Any treatment that the patient received prior to surgery and sample collection;

7 Additional Treatment: Treatment given after initial first round treatment;

8 Tumor Stage: pathological stage of the tumor in AJCC format (Primary Tumor: T; Stage I: IA; IB; IC; Stage II: IIA; IIB; IIC; Stage III: IIIA; IIIB; IIIC; Stage IV: IV);

9 I_II: Stage I or II ovarian cancer;

10 III: Stage III ovarian cancer;

11 IV: Stage IV ovarian cancer;

12 Tumor Grade: Numeric value used to express the degree of abnormality of cancer cells;

13 I or II: Grade I or II tumor;

14 Rest: Any tumor grades other than I or II;

15 Tumor Residual Disease: Measure of the largest remaining nodule;

16 0: No macroscopic disease;

17 1_20∶1–20 mm;

18 >20: Greater than 20 mm;

19 Recurrence: Return of cancer.

The distribution of observations per cohort variable level in the training and validation sets was consistent (**Table 1**). The representation of treatment, preadjuvant therapy, additional treatment, tumor stage, tumor grade, tumor residual disease, and recurrence was comparable between data sets. None of the 15 sample source sites dominated the representation in either training or validation set.

The correlations between the observed and predicted time-to-death and time-to-recurrence were approximately 0.60. Higher correlations (0.8 on average) were observed when only the lower times-to-event were considered because more observations were available and more precise predictions could be obtained. Prediction of longer time-to-event intervals were associated with higher uncertainty due to fewer observations within cohort variable levels, and thus lower correlations between training and validation data sets. The moderate correlation between the two time-to-event analyses suggests the differences in the magnitude and direction of genomic and environmental effects on ovarian cancer survival and recurrence.

### miRNA Biomarkers of Ovarian Cancer Survival and Recurrence

**Tables 2** and **3** list the 16 and 14 miRNAs simultaneously associated with ovarian cancer survival and recurrence detected by the three-step feature selection approach and supporting literature references. The vast majority of the miRNAs associated with survival detected in this study have been reported by other studies. This level of validation reaffirms the validity of the approach undertaken and of the results presented. Of the 16 miRNAs associated with survival, 12 miRNAs have been previously associated with ovarian cancer (hsa-miR-144, hsa-miR-16, hsa-miR-182*, hsa-miR-521, hsa-miR-18b*, hsa-miR-19a*, hsa-miR-22*, hsa-miR-381, hsa-miR-485-3p, hsa-miR-509-3-5p, hsa-miR-148a, and hsa-miR-106b) and one miRNA has been associated with cervical cancer (hsa-miR-329; [23]. The literature review supporting these results was summarized in **Table 2**.

**Table 2:** MicroRNAs associated with post-diagnostic survival and supporting independent studies.

MicroRNA	*P*-Value	Estimate	Hazard Ratio (95% C.I.^1^)	Relevant Literature References
hsa-miR-22*	<.0001	−1.4007	0.25 (0.14 to 0.44)	[24], [25], [30], [33], [36] ^O^
hsa-miR-770-5p	<.0001	−1.2946	0.27 (0.16 to 0.47)	NA
hsa-miR-485-3p	<.0001	−0.8158	0.44 (0.30 to 0.66)	[28] ^O^
hsa-miR-16	<.0001	0.7249	2.07 (1.53 to 2.79)	[24], [25], [27] ^O^
hsa-miR-144	<.0001	0.2644	1.3 (1.14 to 1.49)	[24] ^O^
ebv-miR-BHRF1-2*	0.0001	1.4787	4.39 (2.06 to 9.33)	NA^2^
hsa-miR-182*	0.0001	0.8547	2.35 (1.51 to 3.65)	[24], [25], [72], [73] ^O^
hsa-miR-381	0.0001	0.6801	1.97 (1.40 to 2.79)	[31] ^O^
hsa-miR-509-3-5p	0.0001	−0.3725	0.69 (0.57 to 0.83)	[74] ^O^
hsa-miR-19a*	0.0002	0.5574	1.75 (1.31 to 2.33)	[24] ^O^
hsa-miR-573	0.0007	0.6298	1.88 (1.31 to 2.70)	NA
hsa-miR-329	0.0031	−1.4082	0.25 (0.10 to 0.62)	[23] ^Z^
hsa-miR-106b	0.0024	0.4525	1.57 (1.17 to 2.11)	[24], [25] ^O^
hsa-miR-18b*	0.0042	0.6678	1.95 (1.24 to 3.08)	[24], [26] ^O^
hsa-miR-521	0.0051	1.3416	I_II^3^ = 2.10 (0.89 to 4.97)	[30] ^O^
			Rest^4^ = 0.55 (0.40 to 0.76)	
hsa-miR-148a	0.0063	−0.2493	0.78 (0.65 to 0.93)	[28] ^O^

1 C.I.: Confidence Interval;

2 NA: No information found; ^O^Associated with Ovarian Cancer; ^Z^Associated with other cancer type;

3 I_II: Grade I or II tumor;

4 Rest: Any tumor grades other than I or II.

**Table 3:** MicroRNAs associated with post-diagnostic recurrence on a cohort-independent or -dependent manner and supporting independent studies.

MicroRNA	*P*-Value	Estimate	Hazard Ratio (95% C.I.^1^)	Relevant Literature References
hsa-miR-550*	<.0001	−2.1165	0.12 (0.05 to 0.29)	NA
hsa-miR-22*	<.0001	−1.4397	0.24 (0.12 to 0.46)	[25], [33], [36], [75] O
hsa-miR-223	<.0001	0.5267	1.69 (1.36 to 2.12)	[33], [36], [75] O
hsa-miR-146a	<.0001	−0.4869	0.62 (0.49 to 0.77)	[75] O
hsa-miR-497	0.0001	1.5869	Chemo^3^ = 0.84 (0.69 to 1.03)	[34], [35] O
		1.125	C_O^4^ = 0.53 (0.20 to 1.41)	
			Other^5^ = 0.17 (0.08 to 0.35)	
hsa-miR-214*	0.0001	0.7059	2.03 (1.41 to 2.91)	[2], [25], [36], [75] O
ebv-miR-BHRF1-2*	0.0028	1.092	2.98 (1.46 to 6.10)	NA
hsa-miR-96	0.0065	0.1984	1.22 (1.06 to 1.41)	[26], [34], [37], [75] O
hsa-miR-924	0.0102	1.3019	3.68 (1.36 to 9.92)	NA
hsa-miR-28-3p	0.0109	1.1811	3.26 (1.31 to 8.09)	NA^2^
hsa-miR-369-3p	0.0130	0.4208	1.52 (1.09 to 2.12)	[38] Z

1 C.I.: Confidence Interval;

2 NA: No information found;

O Associated with Ovarian Cancer;

Z Associated with other cancer type;

3 Chemo: Only chemotherapy;

4 C_O: Chemotherapy plus another therapy;

5 Other: Any therapy other than chemotherapy.

Of the miRNAs previously associated with ovarian cancer, the trends of all 12 miRNA were consistent with those reported in previous studies. Hsa-miR-144 (HR = 1.30), hsa-miR-16 (HR = 2.07), hsa-miR-182* (HR = 2.35), hsa-miR-18b* (HR = 1.95), hsa-miR-19a* (HR = 1.75), and hsa-miR-106b (HR = 1.57) were over-expressed in all 3 ovarian tumor histologic subtypes relative to normal primary human ovarian surface epithelium cultures [24]. The consistency between the detected and previously reported trends further supports the biomarker detection strategy presented.

Hsa-miR-182 was also up-regulated in ovarian carcinoma in Stage III/IV epithelial ovarian carcinoma versus normal tissue [25] and has been associated with higher death hazard in glioblastoma multiforme patients receiving chemotherapy plus radiation and targeted treatment [8]. The region containing hsa-miR-182 was amplified in 28.9% of the epithelial ovarian cancer, implying an oncogene-type function, and possibly targets genes forkhead box O1, forkhead box O3 (*FOXO1*;*FOXO3*) which are involved in promoting differentiation and grown inhibition (tumor suppressors,(25)). Hsa-miR-18b* and hsa-miR-16 were found to robustly distinguish ovarian cancer tumors from normal tissue and were significantly up-regulated in ovarian cancer [26]. Hsa-miR-16 (HR = 2.07) has been shown to be up-regulated in serous ovarian carcinoma versus normal ovarian tissues, as well as up-regulated in stage III/IV ovarian cancer versus normal ovarian tissue [25], [27]. Hsa-miR-22 (HR = 0.25) was under-expressed in 3 ovarian tumor histologic subtypes relative to normal primary human ovarian surface epithelium cultures [24]. Hsa-miR-22 was also down -regulated in Stage III/IV epithelial ovarian carcinoma versus normal and up-regulated in primary versus recurrent serous papillary ovarian carcinomas [25]. Hsa-miR-148a (HR = 0.78) was down-regulated in ovarian cancer cell lines and may be involved in the carcinogenesis of ovarian cancer through deregulation of cell proliferation [28]. Hsa-miR-509-3-5p (HR = 0.69) was over-expressed in stage I ovarian cancer relative to stage III ovarian cancer with a p-value = 0.017 and fold-change = 4.01 [29]. Both, hsa-miR-521 and hsa-miR-381 were over-expressed in platinum resistant versus platinum sensitive ovarian cancer [30], [31].

**Figure 1:**
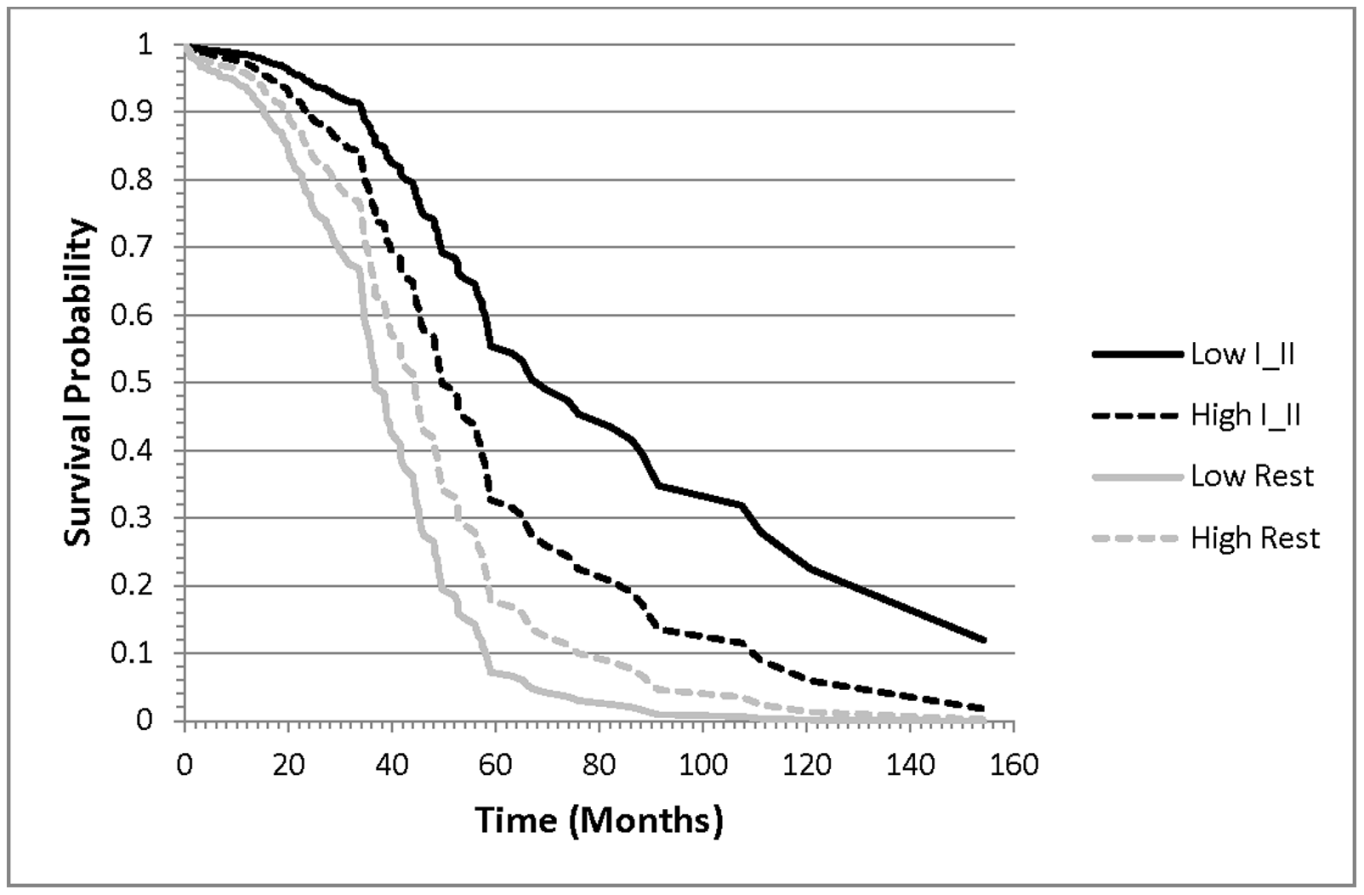
Probability of ovarian cancer survival for patients that have lower grade (I and II) tumors (black lines) or higher (Rest) grade tumors (gray lines) and high (dashed lines) or low (solid line) levels of hsa-miR-521.

The evaluation of clinical factor dependent associations between miRNAs and ovarian cancer survival offer insights into general and condition-specific biomarkers. Of the 16 miRNAs associated with ovarian cancer survival, 15 exhibited general (clinically independent) associations with survival, meanwhile hsa-miR-521 had a tumor grade-dependent association with survival. The hazard of ovarian cancer death increased 2.10 per unit increase in hsa-miR-521 level in patients that have grade I or II tumors and decreased 0.55 per unit increase in the miRNA in patients that have higher level tumors. The survival plot in **Figure 1** depicts the association between the probability of ovarian cancer survival and the interaction between miRNA expression and tumor grade. Lower expression of hsa-miR-521 was associated with the lowest and highest probability of survival in the presence of high (Rest) and low (I and II) grade tumors, respectively.

Similarly to the findings for survival, the majority of the miRNAs associated with recurrence have been previously associated with ovarian cancer, thus reaffirming the reliability of the feature selection approach implemented. Among the 14 miRNAs associated with recurrence in ovarian cancer (**Table 3**), 9 have been previously linked to ovarian cancer (hsa-miR-146a, hsa-miR-15b, hsa-miR-16, hsa-miR-206, hsa-miR-214*, hsa-miR-22*, hsa-miR-223, hsa-miR-497, and hsa-miR-96), and one had a previous association with paired lung primary tumors (hsa-miR-369-3p). **Table 3** summarizes the literature review supporting the detected associations.

The trends of the 9 miRNA previously linked to ovarian cancer and also found in this study were consistent with previously reported. Hsa-miR-146a (HR = 0.62) was under-expressed in ovarian tumor histologic subtypes relative to normal primary human ovarian surface epithelium cultures [24]. Hsa-miR-206 (HR = 0.59) was down-regulated in ovarian cancer cell lines versus normal [32]. Hsa-miR-22 (HR = 0.24) was over-expressed in recurrent ovarian cancer versus primary ovarian cancer [33]. This miRNA also was down-regulated in ovarian carcinoma in early stage versus late stage; down-regulated in Stage III/IV epithelial ovarian carcinoma versus normal; and up-regulated in primary versus recurrent serous papillary ovarian carcinomas [25]. Hsa-miR-497 (in this study HR Chemo = 0.84; Chemo_Other = 0.53; Other = 0.17) was down-regulated in ovarian cancer cell line versus normal ovarian cell lines [34], [35]. Hsa-miR-16 (HR = 2.76) up-regulated in serous ovarian carcinoma versus normal ovarian tissues, as well as up-regulated in stage III/IV ovarian cancer versus normal ovarian tissue as well [25]–[27]. Hsa-miR-214 (HR = 2.03) was over-expressed ovarian tumor histologic subtypes relative to normal primary human ovarian surface epithelium cultures [24]. In a study of epithelial ovarian cancer, hsa-miR-214 was differentially expressed in those with recurrence compared with those without recurrence in both a training and validation set. Tumor tissue samples from those with recurrence were up-regulated compared with those without recurrence in epithelial ovarian cancer [36]. Hsa-miR-214 expression was associated with high grade and late stage tumors, was up-regulated in ovarian cancer tumor tissues, and has a potential role in recurrence [25]. Hsa-miR-214 was also found to play a role in ovarian cancer by targeting PTEN [2].

Hsa-miR-223 (HR = 1.69) was over-expressed in all 3 ovarian tumor histologic subtypes relative to normal primary human ovarian surface epithelium cultures [24]. Hsa-miR-223 was over-expressed in recurrent ovarian cancer versus primary ovarian cancer [33]. Hsa-miR-223 was up-regulated in tumor tissue sample from those with recurrence compared with those without recurrence in epithelial ovarian cancer [36]. Hsa-miR-96 (HR = 1.22) was over-expressed in ovarian cancer cell lines versus normal ovarian cell lines [24], [26], [34], [37]. Hsa-miR-369-3p (HR = 1.52), associated with ovarian cancer recurrence in this study, was similarly up-regulated in paired lung primary tumors [38].

**Figure 2:**
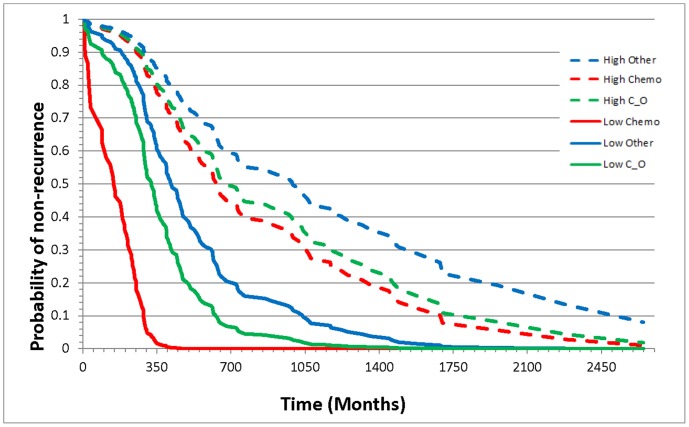
Probability of ovarian cancer non-recurrence for patients receiving the treatment chemotherapy only, chemotherapy along with another treatment, or some other treatment or combination of treatments except chemotherapy that have high or low levels of hsa-miR-497.

The study of interactions between miRNA expression and cohort factors supported the identification of individualized biomarkers. General associations between miRNAs and recurrence irrespective of cohort factors were identified for 13 miRNA. A treatment-dependent association between risk or hazard of recurrence and hsa-miR-497 was identified. The hazard for ovarian cancer recurrence decreased with increasing miRNA level in patients for all three treatments (Chemo, Chemo_Other, Other), and the hazard was lowest (0.17) for individuals receiving the Other treatment. **Figure 2** depicts the association between the probability of non-recurrence interaction between level of hsa-miR-497 and treatment. The probability of non-recurrence was distinctively lower in patients with low miRNA levels receiving Chem treatment, however was not different between patients receiving Chemo or Chemo and Other treatments when the levels of miRNA were high.

### Transcription Factors and Target Genes Associated with Survival and Recurrence

In total, the expression of 838 and 734 target genes and 12 and eight TFs were associated (FDR-adjusted P-value <0.05) with ovarian cancer survival and recurrence, respectively. The TFs associated with ovarian cancer survival and recurrence and supporting literature review are listed in **Tables 4** and **5**, respectively.

**Table 4:** Transcription factors associated with ovarian cancer survival.

Transcription Factor	Estimate	Hazard Ratio (95% C.I.^1^)	Relevant Literature References
*CLOCK*	0.0097	0.81 (0.58 to 1.11)	[56], [57] Z
*EGR1*	0.0065	1.15 (1.03 to 1.28)	[39] Z
*EGR2*	0.0038	1.17 (1.06 to 1.30)	[40], [76] Z
*ESR2*	0.0065	0.66 (0.49 to 0.88)	[49]–[51] O
*ETS2*	0.0098	1.32 (0.99 to 1.76)	[50], [53] Z
*FOS*	0.0056	1.15 (1.03 to 1.28)	[77] O
*HDAC3*	0.0093	1.63 (1.13 to 2.37)	[58] O
*HOXA1*	0.0096	0.71 (0.53 to 0.97)	[59] Z
*MYC*	0.009	1.27 (1.05 to 1.54)	[46], [47], [78] O
*NR5A1*	0.0086	0.53 (0.34 to 0.82)	[55] O
*POU2F2*	0.008	0.64 (0.45 to 0.93)	[52] Z
*TGFB1*	0.0054	0.46 (0.32 to 0.66)	[42]–[45] O

1 C.I.: Confidence Interval;

2 NA: No information found;

O Associated with Ovarian Cancer;

Z Associated with other cancer type.

**Table 5:** Transcription factors associated with ovarian cancer recurrence.

Transcription Factor	Estimate	Hazard Ratio (95% C.I.^1^)	Relevant Literature References
*CTCF*	0.0063	1.71 (1.11 to 2.64)	[60], [61] Z
*EGR1*	0.0076	1.15 (1.03 to 1.28)	[39] Z
*EGR2*	0.0054	1.17 (1.05 to 1.31)	[40], [76] Z
*FOS*	0.0092	1.13 (1.01 to 1.27)	[41] O
*MYOD1*	0.0075	0.77 (0.63 to 0.95)	[62], [63] Z
*SOX18*	0.0082	0.77 (0.62 to 0.95)	[64], [65] Z
*TBP*	0.0088	1.63 (1.19 to 2.24)	[66] Z
*TGFB1*	0.0088	0.56 (0.39 to 0.80)	[42]–[45] O

1 C.I.: Confidence Interval;

O Associated with Ovarian Cancer;

Z Associated with other cancer type.

The four TFs significantly associated with both ovarian cancer survival and recurrence (early growth response 1 (*EGR1*), early growth response (*EGR2*), FBJ murine osteosarcoma viral oncogene homolog (*FOS*), and transforming growth factor beta 1(*TGFB1*), exhibited trends consistent with previous studies. *EGR1* (HR = 1.15 for survival and recurrence) has a key role in carcinogenesis and cancer recurrence, and exhibits increased expression in gastric cancer tissues relative to normal mucosa [39]. The positive association between *EGR2* and hazard uncovered in this study (HR = 1.17 for survival and recurrence) was confirmed with reports that this TF plays a key role in the PTEN-induced apoptotic pathway. Furthermore, studies suggest that this TF may be a promising target molecule for gene therapy to treat a variety of cancers [40]. *FOS* expression (HR = 1.15; 1.13 for death and recurrence in this study, respectively) has been associated with ovarian cancer, and is a molecular predictor of recurrence and survival in epithelial ovarian carcinomas [41]. *TGFB1* (HR = 0.46; 0.56 for death and recurrence in this study, respectively) has been linked to ovarian cancer [42]–[44], and may play an important role in ovarian cancer biology with potential effects on tumor growth and angiogenesis [45].

#### Transcription factors associated with survival

Eight TFs were solely associated with the hazard of ovarian cancer death: circadian locomotor output cycles kaput (*CLOCK*), estrogen receptor 2 (*ESR2*), v-ets erythroblastosis virus E26 oncogene homolog 2 (*ETS2*), histone deacetylase 3 (*HDAC3*), homeobox A1 (*HOXA1*), v-myc myelocytomatosis viral oncogene homolog (*MYC*), nuclear receptor subfamily 5, group A, Member 1 (*NR5A1*), and POU class 2 homeobox 2 (*POU2F2*), and their trends were in agreement with previous studies. *MYC* (HR = 1.27) contributes independently to ovarian and breast pathogenesis when over-expressed [46], and was more frequently detected in malignant ovarian tumors when compared with benign ovarian tumors [47], [48]. *ESR2* (HR = 0.66) was significantly lower in ovarian cancer cell lines and tissues than in their corresponding normal counterparts [49]. *ESR2* has been associated with malignant ovarian epithelial cells [50] and may be a susceptibility marker for epithelial ovarian cancer [51].

The opposite association between *POU2F2* expression and ovarian cancer hazard (HR = 0.64) detected in this study was consistent with reports that this TF, a member of the POU homeodomain family of transcriptional regulators critical for normal embryonic development, was associated with down-regulation of B-cell CLL/lymphoma 2 (*BCL-2*) that results in apoptosis [52]. Over-expression of *ETS2* has previously been shown in human esophageal squamous cell carcinoma and breast cancer [50], [53]. This TF also plays a role in regulation of the production of TF *MYC*, also significantly associated with increased hazard (HR = 1.27) in ovarian cancer in this study [54]. These findings were in agreement with the positive association between *ETS2* and ovarian cancer death hazard detected in this study (HR = 1.32). Defects in *NR5A1* (HR = 0.53 in this study) can result in arrest of ovarian function [55]. The relationship between *CLOCK* and ovarian cancer survival detected in the present study (HR = 0.81) agrees with the report that variations in the epigenetics of *CLOCK* may lead to increased risk of breast cancer [56], and that in women with breast cancer, there was significantly less methylation of the *CLOCK* promoter region [57]. Similar to this study, *HDAC* (HR = 1.63) was over-expressed in 80% of cases of ovarian cancer, with no significant difference in the expression profiles between histological subtypes [58]. Suppression of *HOXA1* (HR = 0.71) has been linked to an increase of invasive cancer cells in human pancreatic cancer [59].

#### Transcription factors associated with recurrence

Four TFs were only associated with the hazard of ovarian cancer recurrence, and their trends were all consistent with previous work: CCCTC-binding factor (*CTCF*), Myogenic Differentiation 1(*MYOD1*), SRY (sex determining region Y)-Box 18 (*SOX18*), and TATA Box Binding Protein (*TBP*). *CTCF* (HR = 1.71) plays a role in breast cancer [60], [61], and *MYOD1 (*HR = 0.77) has previously been associated with cervical cancer [62], [63]. *SOX18* (HR = 0.77), a member of the SOX family of transcription factors involved in the determination of the cell fate, has been proposed as a useful target for human cancer treatment [64], [65]. Consistent with our findings on ovarian cancer, *TBP* (HR = 1.63), which is highly expressed in the ovary, has elevated expression in human colon carcinomas [66].

#### Target gene biomarkers of ovarian cancer survival and recurrence

Among the target genes associated with ovarian cancer survival, 16 were identified in the Dragon database of ovarian cancer genes: acetylcholinesterase (*ACHE*); BCL2-antagonist/killer 1 (*BAK1*); B-cell CLL/lymphoma 2 (*BCL2*); CD44 molecule (Indian blood group) (*CD44*); CD63 molecule (*CD63*); cadherin 13, H-cadherin (heart) (*CDH13*); cyclin-dependent kinase inhibitor 2B (p15, inhibits CDK4) (*CDKN2B*); colony stimulating factor 1 receptor (*CSF1R*); cathepsin D (*CTSD*); discoidin domain receptor tyrosine kinase 1 (*DDR1*); galactose-1-phosphate uridylyltransferase (*GALT*); kallikrein-related peptidase 9 (*KLK9*); mitogen-activated protein kinase kinase 1 (*MAP2K1*); mitogen-activated protein kinase kinase 3 (*MAP3K3*); *MYC*, and platelet-derived growth factor receptor, alpha polypeptide (*PDGFRA*). Likewise, among the genes associated with ovarian cancer recurrence, 9 were identified in the Dragon database: *ACHE*; *BAK1*; breast cancer 1, early onset (*BRCA1* **)**; *CD44*; *CTSD*; *DDR1*; *KLK9*; antigen identified by monoclonal antibody Ki-67 (*MKI67*), and topoisomerase (DNA) II alpha 170 kDa (*TOP2A*).

#### Validation

Two indicators of the reliability of the predictive profiles in the independent validation were considered. The relative increment in MSE of the model including the cohort-independent and dependent-expression profiles between the training and validation data set were 13.4% and 15.4% for survival and recurrence, respectively. As expected, the predictive equation offered a better description of the data used to develop the equation (i.e. the training data set), and a small difference between training and validating data was expected due to sampling effects such as between-patient variation. The small increase in MSE between the training and validating data set was a first, global indicator of the similar profile-hazard relationship identified in both independent data sets and of the replicability of our findings. Second, the Pearson (and Spearman) correlations of the profile associations with death and recurrence between the training (e.g. Tables 2, 3, 4, 5) and validation data sets were 89.7% (84.5%) and 87.3% (82.4%), respectively. The cross-validation results and the agreement between the literature review and the present findings further suggest that the detected profiles associated with ovarian cancer are likely to be replicable. Experimental confirmation of the findings is needed.

### Functional Gene Groups Associated with Ovarian Cancer Survival and Recurrence

The functional analyses of the target genes associated with ovarian cancer uncovered enriched pathways and processes, many of which were previously associated with ovarian cancer. Analysis of the significant target genes associated with ovarian cancer survival using a Fisher exact test uncovered enrichment of biological processes including ribonucleotide biosynthetic process (P-value <0.002, 14 genes) and immune response (P-value <0.0004, 49 genes) and the KEGG pathways lysosome (P-value <0.001, genes15) and epithelial cell signaling (P-value <0.001, genes 11). Likewise, analysis of the significant target genes associated with ovarian cancer recurrence using a Fisher exact test uncovered enrichment of biological processes including the NAD metabolic process (P-value <0.001, 6 genes), M phase (P-value <0.002, 24 genes), and pyrimidine-and nicotineamide-nuclotide metabolic processing (P-value <0.02, 6 genes).

The set enrichment analysis of all target genes segmented by their positive or negative association with survival or recurrence offered additional insights into the functional categories differentially represented among gene groups. **Table 6** lists the GO biological processes differentially (FDR-adjusted P-value <0.05, >75 genes) represented between the genes that have negative or positive associations with death and recurrence hazard. Two GO biological processes had significant differential enrichment between the genes segmented by low and high hazard of ovarian cancer death. Likewise, 12 GO biological processes had significant differential enrichment between the genes associated with low and high hazard of ovarian cancer recurrence. **Table 6** includes the corresponding characterization of the differential enrichment (log_e_(odds ratio)), and the statistical significance level. A log_e_(odds ratio) >0 (<0) indicates that the category was more (less) enriched among the genes with lower hazard relative to the genes with higher hazard of death or recurrence. Among the significant categories, all were characterized by log_e_(odds ratio) >0, indicating that there were more genes pertaining to the category in the low hazard group relative to the high hazard group. **Figures S1** and **S2** depict the relation between the GO biological processes associated with the hazards of ovarian cancer death and recurrence inferred from the set enrichment analysis, respectively. Processes associated with general metabolism were differentially enriched among the genes associated with ovarian cancer death. Processes associated with nucleotide metabolism and transcription were enriched among the genes associated with ovarian cancer survival recurrence. The processes identified by the set enrichment analyses were consistent with the results from the significant gene list enrichment analyses. The results from our functional analyses of target genes associated with ovarian cancer survival and recurrence were in agreement with previous studies. Transcriptome analysis has shown that suppression of NOTCH signaling in ovarian cancer cells led to down-regulation of genes in pathways involved in cell-cycle regulation and nucleotide metabolism [67]. The inhibition of cell proliferation in an ovarian cancer cell line in response to a differentiation-inducing agent was related to a shift in the direction of the purine metabolism from anabolism to catabolism [68]. Inhibition of cell metabolism has been proposed as an effective treatment against human epithelial ovarian carcinomas [69].

**Table 6:** Differentially enriched Gene Ontology biological processes among all target genes segmented by low and high hazard of ovarian cancer death or recurrence identified by set enrichment analyses.

		− hazard genes^1^		+ hazard genes^2^		Log_e_ 3	FDR-
Trait and GO Category	GO identifier	In GO	Not in GO	In GO	Not in GO	(odds ratio)	adjusted P-value^4^
**Survival**							
regulation of cellular metabolic process	GO:0031323	218	502	198	766	0.52	1.46E−02
regulation of metabolic process	GO:0019222	229	491	214	750	0.49	1.47E−02
**Recurrence**							
nucleobase, nucleoside, nucleotide and nucleic acid metabolic process	GO:0006139	117	175	321	1034	0.77	3.91E−05
RNA processing	GO:0006396	29	263	49	1306	1.08	5.96E−03
nitrogen compound metabolic process	GO:0006807	122	170	354	1001	0.71	1.94E−04
regulation of gene expression	GO:0010468	84	208	237	1118	0.64	5.48E−03
gene expression	GO:0010467	109	183	291	1064	0.78	3.91E−05
regulation of transcription	GO:0045449	78	214	213	1142	0.67	5.48E−03
cellular metabolic process	GO:0044237	264	223	492	668	0.47	8.33E−03
cellular biosynthetic process	GO:0044249	106	186	342	1013	0.52	3.13E−02
RNA metabolic process	GO:0016070	80	212	219	1136	0.67	5.48E−03
transcription	GO:0006350	82	210	219	1136	0.71	3.22E−03
regulation of nitrogen compound metabolic process	GO:0051171	84	208	237	1118	0.64	5.48E−03
macromolecule biosynthetic process	GO:0009059	97	195	283	1072	0.63	5.42E−03

1 − hazard genes: number of genes that have a negative association between the hazard of ovarian cancer death (higher survival) or recurrence and expression.

2 + hazard genes: number of genes that have a positive association between the hazard of ovarian cancer death (lower survival) or recurrence and expression.

3 Log_e_(Odds Ratio): values >1 indicate that the category was more enriched among the genes that have a negative association with hazard than among the genes that have a positive association with hazard; values <1 indicate that the category was more enriched among the genes that have a positive association with hazard than among the genes that have a negative association with hazard; Extreme values indicate higher difference in the enrichment percentages between the negative and positive association groups. Values close to zero indicate similar enrichment percentages between positive and negative association groups.

4 FDR-adjusted P-value: False discovery rate adjusted P-value of the log odds ratio test. Enrichment at FDR-adjusted Pvalue <0.05) and ≥75 genes in the category.

### microRNA-transcription Factor-target Gene Networks of Survival and Recurrence

In gene regulatory networks, TFs and miRNAs regulate each other and the expression of target genes [70]. The binding sites of TFs and genes can be the target of miRNAs and other TFs. Transcription factors regulate genes at the DNA level, while miRNAs regulate gene expression post-transcriptionally [14]. Applying previously advocated approaches, this study combined TF and miRNA target prediction together with context-linked (cohort) information and experimental genome-wide co-expression data to identify biologically meaningful molecular interactions [14], [71]. The networks of TFs, miRNAs, and target genes significantly associated with survival or recurrence (P-value <0.01), were reconstructed using Cytoscape. The reconstructed molecular networks that integrate the pattern of association between TFs, miRNAs, target genes and survival or recurrence aid in the identification of robust network biomarkers of ovarian cancer.

**Figure 3:**
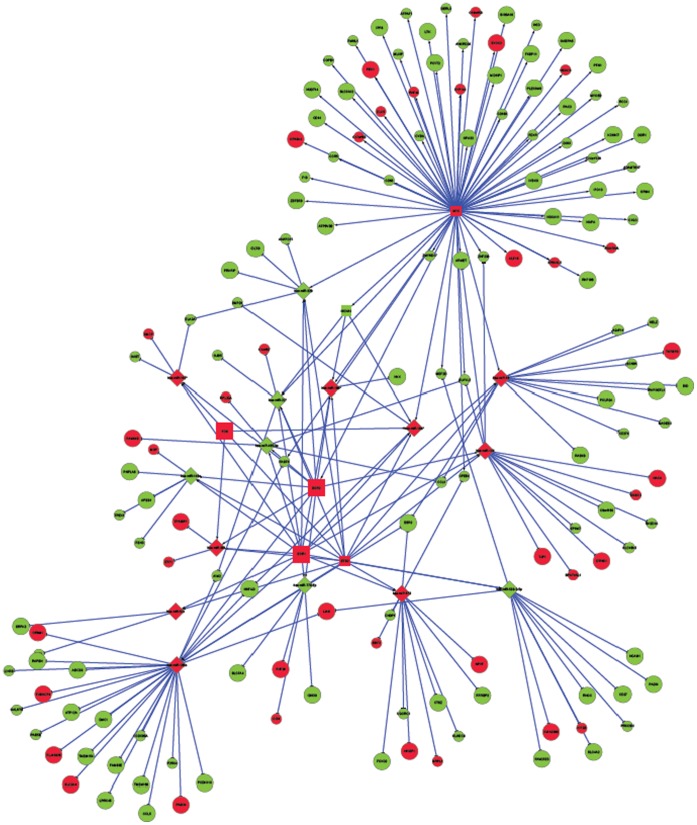
Network of microRNAs, transcription factors, and target genes associated with survival in ovarian cancer. (Node Shape: microRNA = diamond, target gene = circle, transcription factor = square; Node Color: Red indicates increased hazard with high expression, Green indicates decreased hazard with high expression; Node Size: larger indicates a more extreme association (P-value <0.006), smaller indicates a less extreme association.).

**Figure 4:**
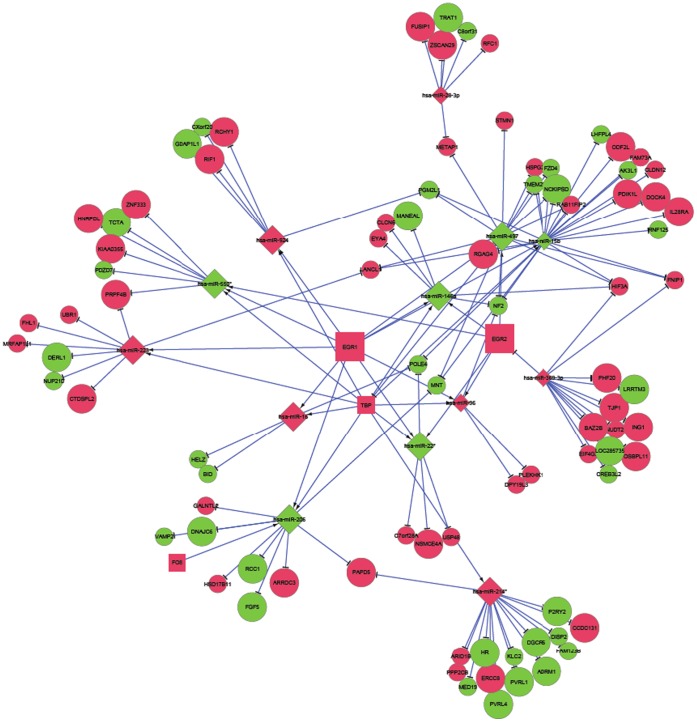
Network of microRNA, transcription factors, and target genes associated with ovarian cancer recurrence. (Node Shape: microRNA = diamond, target gene = circle, transcription factor = square; Node Color: Red indicates increased hazard with high expression, Green indicates decreased hazard with high expression; Node Size: larger indicates a more extreme association (P-value <0.006), smaller indicates a less extreme association.).

**Figures 3** and **4** depict a global network of the miRNAs, TFs and target genes (irrespective of significance) for ovarian cancer survival and recurrence, respectively. These general networks include six and four TFs, 15 and 13 miRNAs and 167 and 89 target genes associated with survival and recurrence, respectively. **Figures S3** and **S4** depict local sub-networks of significant miRNAs, TFs, and targets all significantly associated with ovarian cancer survival and recurrence, respectively. These targeted networks include six and three TFs, 14 and 13 miRNAs and 71 and 56 target genes associated with survival and recurrence, respectively.

The difference in topology between global networks offers insights into the most effective therapies to ameliorate both phenotypes. The networks of survival and recurrence differ in interconnectivity and relation between driver and passenger genomic units. The survival network includes a larger number of driver TFs and miRNAs and a much larger number of target genes that translates into a more driver-centric connectivity than in the recurrence network. Up-regulated miRNAs (red nodes indicate higher hazard of death) appear to dominate as hubs in the survival network, meanwhile a similar number of up and down-regulated miRNAs were hubs in the recurrence network. There were more up than down-regulated TFs in the survival network and no down-regulated TFs in the recurrence network.

For the general and targeted survival networks, four edges was the most frequent shortest path length and was near double the number of paths of length two or three. For the general recurrence networks, four edges was the most frequent shortest path length and was near triple the number of paths of length two or three, meanwhile the distribution of path length was fairly uniform from two to six edges. This pathway comparison indicates that the connections between miRNAs, TFs and target genes were more direct for survival than for recurrence. This result was consistent with the distribution of shared neighbors and average neighborhood connectivity. This distribution was dominated by one shared neighbor in both survival and recurrence networks. However, two and three shared neighbors were more common in the recurrence network. The median average neighborhood connectivity was 15 and seven for the general survival and recurrence networks, respectively. Betweeness and closeness centrality measurements confirmed these trends. Also the centralization of the survival network was double that of the recurrence network meanwhile the density of the networks follows an approximately inverse relationship. The more direct connections and higher centrality of the survival network suggest that network-based approaches to prognosticate or predict ovarian cancer survival may be more effective than those for ovarian cancer recurrence.

### Conclusions

This study demonstrated the feasibility to infer reliable miRNA-TF-target gene networks associated with survival and recurrence of ovarian cancer based on the simultaneous analysis of co-expression profiles and consideration of the clinical characteristics of the patients. The expression of three miRNAs (hsa-miR-16, hsa-miR-22*, and ebv-miR-BHRF1-2*), four TFs (*FOS*, *EGR2*, *EGR1*, and *TGFB1*) and 308 genes were associated with the hazard of ovarian cancer survival and recurrence. Both hsa-miR-16 and hsa-miR-22* were previously linked to ovarian cancer and exhibited trends in this study similar to those in independent studies. The expression of TFs *FOS*, *EGR1*, and *EGR2* was positively associated with ovarian cancer hazard, meanwhile the expression of *TGFB1* was negatively associated with the hazard. These overlapping results suggest the importance of these biomarkers in the recurrence of ovarian cancer and are a strong lead for further experimental validation. This study confirmed 19 miRNAs previously associated with ovarian cancer and identified two miRNAs that have previously been associated with other cancer types. Three miRNAs were associated with both ovarian cancer survival and recurrence and 27 miRNAs were associated with only one hazard. Two miRNAs (hsa-miR-521 and hsa-miR-497) were cohort-dependent, while 28 were cohort-independent. Empirical confirmation of these general and cohort-dependent findings could lead to improved prognostic and predictive tools. In total, the expression of 838 and 734 target genes and 12 and eight TFs were associated (FDR-adjusted P-value <0.05) with ovarian cancer survival and recurrence, respectively. Functional analysis highlighted the association between cellular and nucleotide metabolic processes and ovarian cancer. The more direct connections and higher centrality of the miRNAs, TFs and target genes in the survival network suggest that network-based approaches to prognosticate or predict ovarian cancer survival may be more effective than those for ovarian cancer recurrence. The understanding the biology and molecular pathogenesis of ovarian cancer is key to developing improved prognostic indicators and effective therapies.

## Supporting Information

Figure S1 **Relation between the Gene Ontology biological processes associated with ovarian cancer death inferred from the set enrichment analysis.**
(TIF)

Figure S2 **Relation between the Gene Ontology biological processes associated with ovarian cancer recurrence inferred from the set enrichment analysis.**
(TIF)

Figure S3 **Targeted sub-network of microRNAs, transcription factors, and target genes associated with ovarian cancer survival.** (Node Shape: microRNA = diamond, target gene = circle, transcription factor = square; Node Color: Red indicates increased hazard with high expression, Green indicates decreased hazard with high expression; Node Size: larger indicates a more extreme association (HR ≥ |1.6|), smaller indicates a less extreme association.)
(TIF)

Figure S4 **Targeted sub-network of microRNAs, transcription factors, and target genes associated with post-diagnostic recurrence in ovarian cancer.** (Node Shape: microRNA = diamond, target gene = circle, transcription factor = square; Node Color: Red indicates increased hazard with high expression, Green indicates decreased hazard with high expression; Node Size: larger indicates a more extreme association (HR ≥ |1.6|), smaller indicates a less extreme association.)
(TIF)
