# Supplementary figures and images for: Transcription Factor-MicroRNA-Target Gene Networks Associated with Ovarian Cancer Survival and Recurrence
Source: PLoS One. 2013 Mar 12;8(3):e58608. doi: 10.1371/journal.pone.0058608 (PMC3595291; doi:10.1371/journal.pone.0058608)

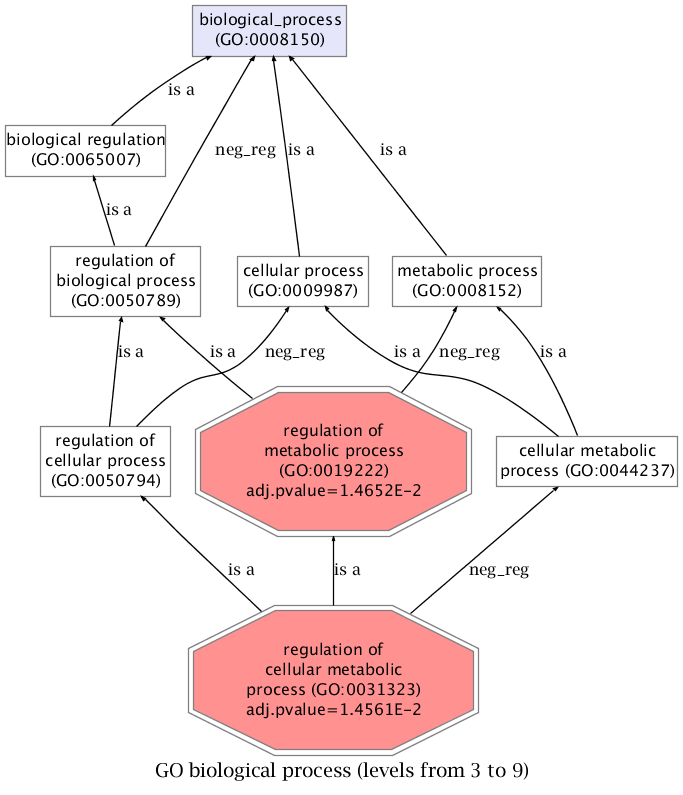

Supplement: Figure S1 — Relation between the Gene Ontology biological processes associated with ovarian cancer death inferred from the set enrichment analysis. (TIF) [file pone.0058608.s001.tif]

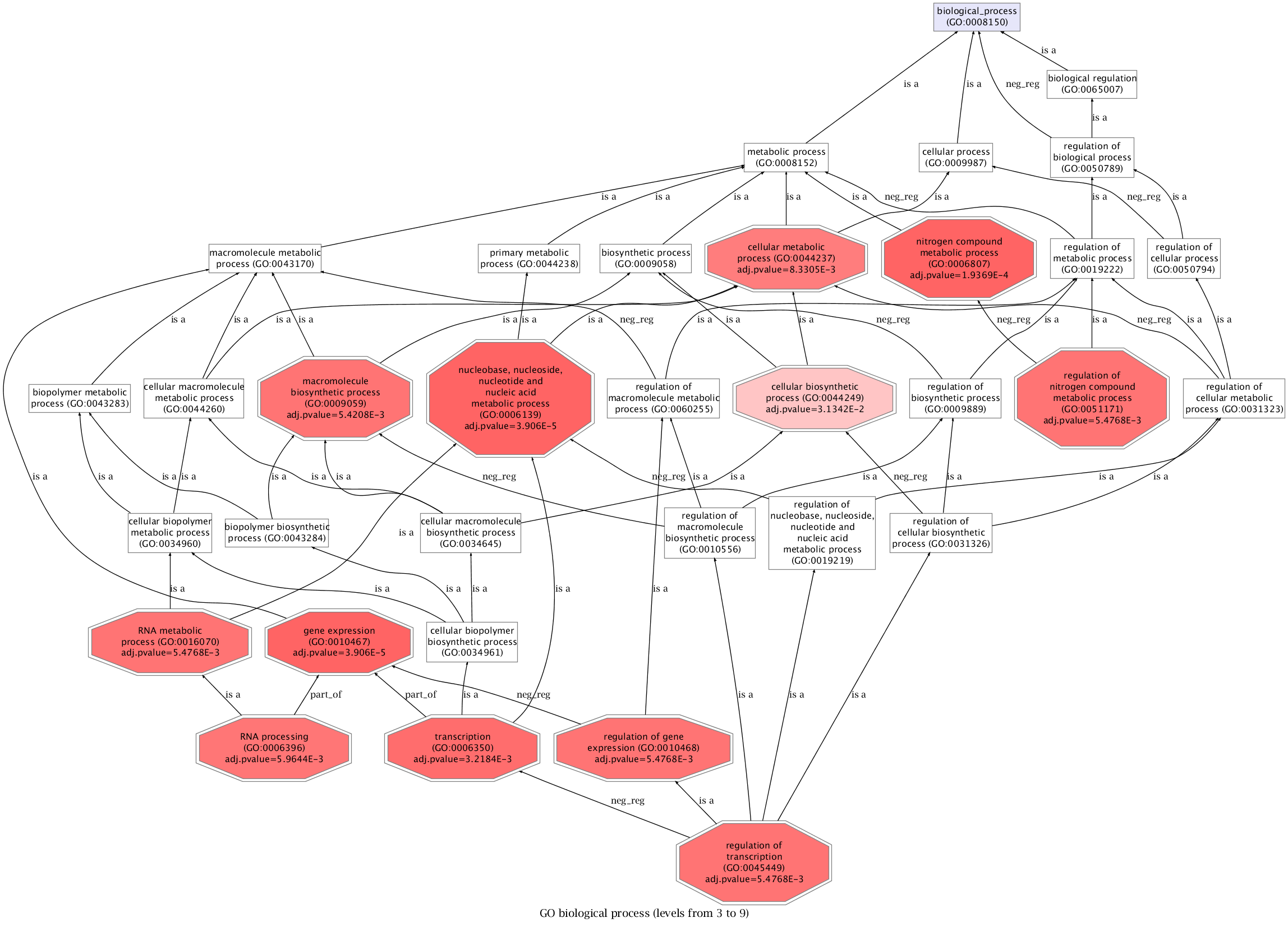

Supplement: Figure S2 — Relation between the Gene Ontology biological processes associated with ovarian cancer recurrence inferred from the set enrichment analysis. (TIF) [file pone.0058608.s002.tif]

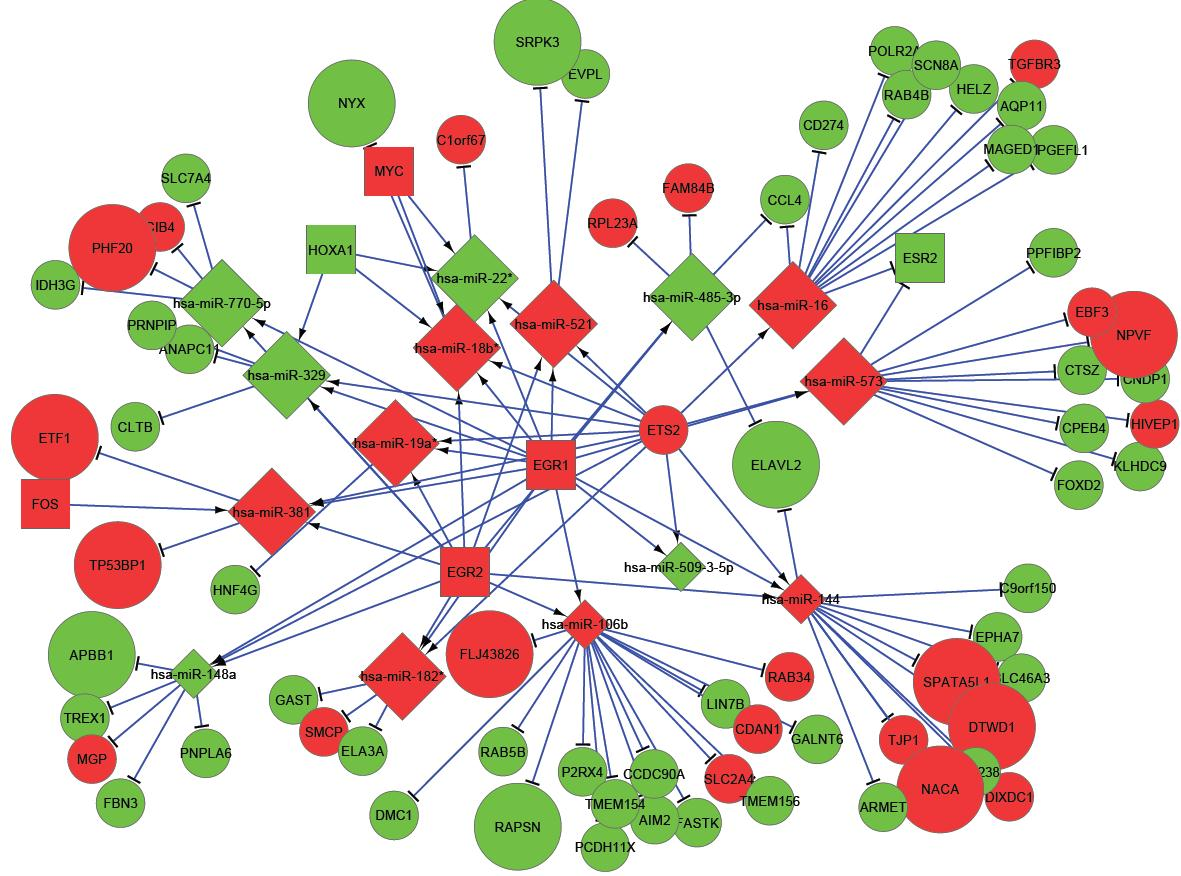

Supplement: Figure S3 — Targeted sub-network of microRNAs, transcription factors, and target genes associated with ovarian cancer survival. (Node Shape: microRNA = diamond, target gene = circle, transcription factor = square; Node Color: Red indicates increased hazard with high expression, Green indicates decreased hazard with high expression; Node Size: larger indicates a more extreme association (HR ≥ |1.6|), smaller indicates a less extreme association.) (TIF) [file pone.0058608.s003.tif]

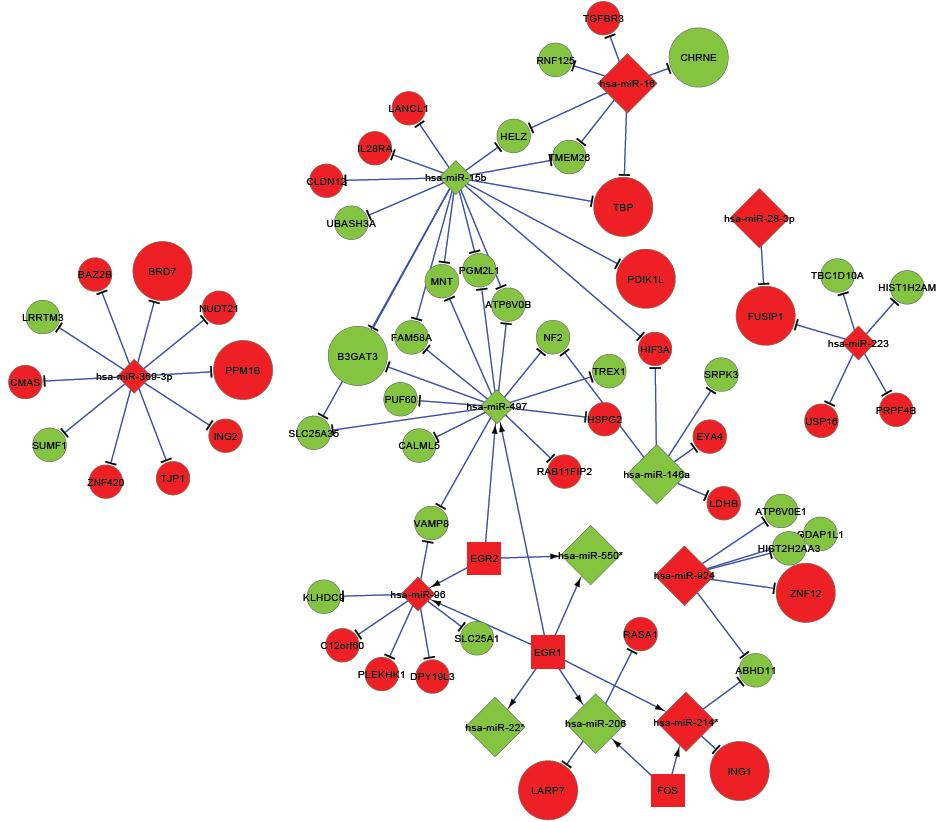

Supplement: Figure S4 — Targeted sub-network of microRNAs, transcription factors, and target genes associated with post-diagnostic recurrence in ovarian cancer. (Node Shape: microRNA = diamond, target gene = circle, transcription factor = square; Node Color: Red indicates increased hazard with high expression, Green indicates decreased hazard with high expression; Node Size: larger indicates a more extreme association (HR ≥ |1.6|), smaller indicates a less extreme association.) (TIF) [file pone.0058608.s004.tif]
